# COVID-19 and Tuberculosis-Related Catastrophic Costs

**DOI:** 10.4269/ajtmh.20-1125

**Published:** 2020-12-02

**Authors:** Ahmad Fuady, Tanja A. J. Houweling, Jan Hendrik Richardus

**Affiliations:** 1 Department of Public Health, Erasmus MC, University Medical Center Rotterdam, Rotterdam, The Netherlands;; 2 Department of Community Medicine, Faculty of Medicine, Universitas Indonesia, Jakarta, Indonesia

## Abstract

The COVID-19 pandemic has created an unprecedented health crisis and a substantial socioeconomic impact. It also affects tuberculosis (TB) control severely worldwide. Interruptions of many TB control programs because of the COVID-19 pandemic could result in significant setbacks. One of the targets that can be affected is the WHO’s End TB Strategy goal to eliminate catastrophic costs of TB-affected households by 2030. Disruptions to TB programs and healthcare services due to COVID-19 could potentially prolong diagnostic delays and worsen TB treatment adherence and outcomes. The economic recession caused by the pandemic could significantly impact household financial capacity because of the reduction of income and the rise in unemployment rates. All of these factors increase the risk of TB incidence and the gravity of economic impact on TB-affected households, and hamper efforts to eliminate catastrophic costs and control TB. Therefore, efforts to eliminate the incidence of TB-affected households facing catastrophic costs will be very challenging. Because financial constraint plays a significant role in TB control, the improvement of health and social protection systems is critical. Even before the pandemic, many TB–high-burden countries (HBCs) lacked robust health and social protection systems. These challenges highlight the substantial need for a more robust engagement of patients and civil society organizations and international support in addressing the consequences of COVID-19 on the control of TB.

## INTRODUCTION

The COVID-19 pandemic has created an unprecedented health crisis, with 15.5 million confirmed cases and over 633,000 deaths worldwide in the first 7 months of the pandemic.^1^ These numbers will continue to increase as the pandemic is predicted to last for 1–2 years.^2^ The pandemic also has substantial consequences for other domains. It has a huge social impact: retracting economic growth and increasing rates of unemployment, poverty, and food insecurity.^3,4^ The pandemic also raises a particular concern for regular healthcare provision as healthcare resources are being shifted to COVID-19–related services^5,6^ and as patients are starting to avoid healthcare services for non-COVID-19–related health problems.^7^

Tuberculosis (TB) control is one of the health domains that are affected by this pandemic.^8^ Before the onset of the COVID-19 pandemic, TB was the single biggest infectious disease killer.^9^ Although the incidence of TB worldwide remains high, that number has remained relatively stable and many countries have even shown significant progress in recent years.^9^ However, because millions of people with TB depend on large-scale TB control programs,^9^ interruptions due to the COVID-19 pandemic could result in significant setbacks.^8^

In 2015, the WHO End TB Strategy set three main milestones for 2020: 20% reduction of TB incidence and 35% reduction of TB mortality compared with 2015, and zero percent of TB-affected households facing catastrophic costs due to TB.^9,10^ The disruptions to TB programs and healthcare services due to COVID-19 could potentially prolong diagnostic delays, increase the rate of undetected TB cases in the community, and leave TB patients untreated, which all would lead to more transmission and TB cases in the upcoming years. If no further action is taken, TB mortality could also increase.^11^

Potential setbacks are also expected in the achievement of the target of eliminating the incidence of catastrophic costs due to TB. Accessing TB-related services has substantial economic consequences, and such consequences can be catastrophic, particularly for poor households.^12,13^ These costs may further reduce household financial capacity and cast them into a poverty trap because catastrophic costs may hamper further access to health care. The COVID-19 pandemic has set an exceptional crisis in a short period and it is expected to affect most people living in poor households. Income loss, rising prices, and limited social protection are expected to push about 71 million people into extreme poverty in 2020 alone.^14^ These figures have been much more severe in low- and middle-income countries (LMICs) that lack strong social protection programs.^15^ It is plausible that accessing TB-related services, therefore, will aggravate this financial burden, and eliminating the incidence of catastrophic costs due to TB will be very challenging.

There has been little discussion on how the COVID-19 pandemic will impact progress toward the elimination of catastrophic costs due to TB. In this article, we describe progress toward the target of eliminating the incidence of catastrophic costs due to TB, the potential impact of COVID-19 on future target achievement, and potential measures that can be implemented to cushion this effect of the pandemic.

## TUBERCULOSIS-RELATED CATASTROPHIC COSTS

In 2015, the WHO End TB Strategy set a new concept of “catastrophic costs,” which is different from “catastrophic expenditures,” a similar-sounding indicator commonly used to measure progress toward universal health coverage (UHC).^16–18^ Whereas catastrophic expenditure focuses on direct medical costs only, TB-related catastrophic costs also include indirect costs because of accessing TB-related services. For the WHO’s End TB Strategy framework, if the total costs incurred by a TB-affected household exceed 20% of household annual income, the costs are defined as catastrophic. Most studies concerning TB-related catastrophic costs have used this definition, using various thresholds in the sensitivity analyses.^19^

The WHO has encouraged many countries to implement a national TB patient costs survey. This is crucial to obtain a clear picture of the financial burden faced by TB-affected households. By July 2019, 17 countries had accomplished the national costs survey, whereas nine countries were underway.^9,20–23^ Thirty-one other countries are preparing survey and expected to collect data in 2020 or 2021. Until now, the national surveys revealed that incidence of TB-related catastrophic costs remains high in many countries, ranging from 19% (95% CI: 15–25) in Lesotho to 83% (95% CI: 80–86) in Timor Leste.^24,25^ Among households affected by multidrug-resistant (MDR) TB, the incidence was consistently higher than among TB-affected households, ranging from 67% (95% CI: 62–72) in the Philippines and 67% (95% CI: 42–85) in Benin to 100% (95% CI: 92–100) in Uganda.^25^ In addition to the national costs surveys, many studies reported the incidence of catastrophic costs at the local level, such as district, province, or several provinces.^13,26–28^ Despite the various methods applied, the studies at the local level also suggest that the incidence of TB-related catastrophic costs remains high.

Looking at the current picture, eliminating the incidence of catastrophic costs remains very challenging. In the current context of the COVID-19 pandemic and global economic recession, the proximate WHO End TB Strategy milestone of zero TB-affected families incurring catastrophic costs by 2025 seems unlikely to be achieved.

## EFFECTS ON TB-RELATED COSTS

**Figure 1.**
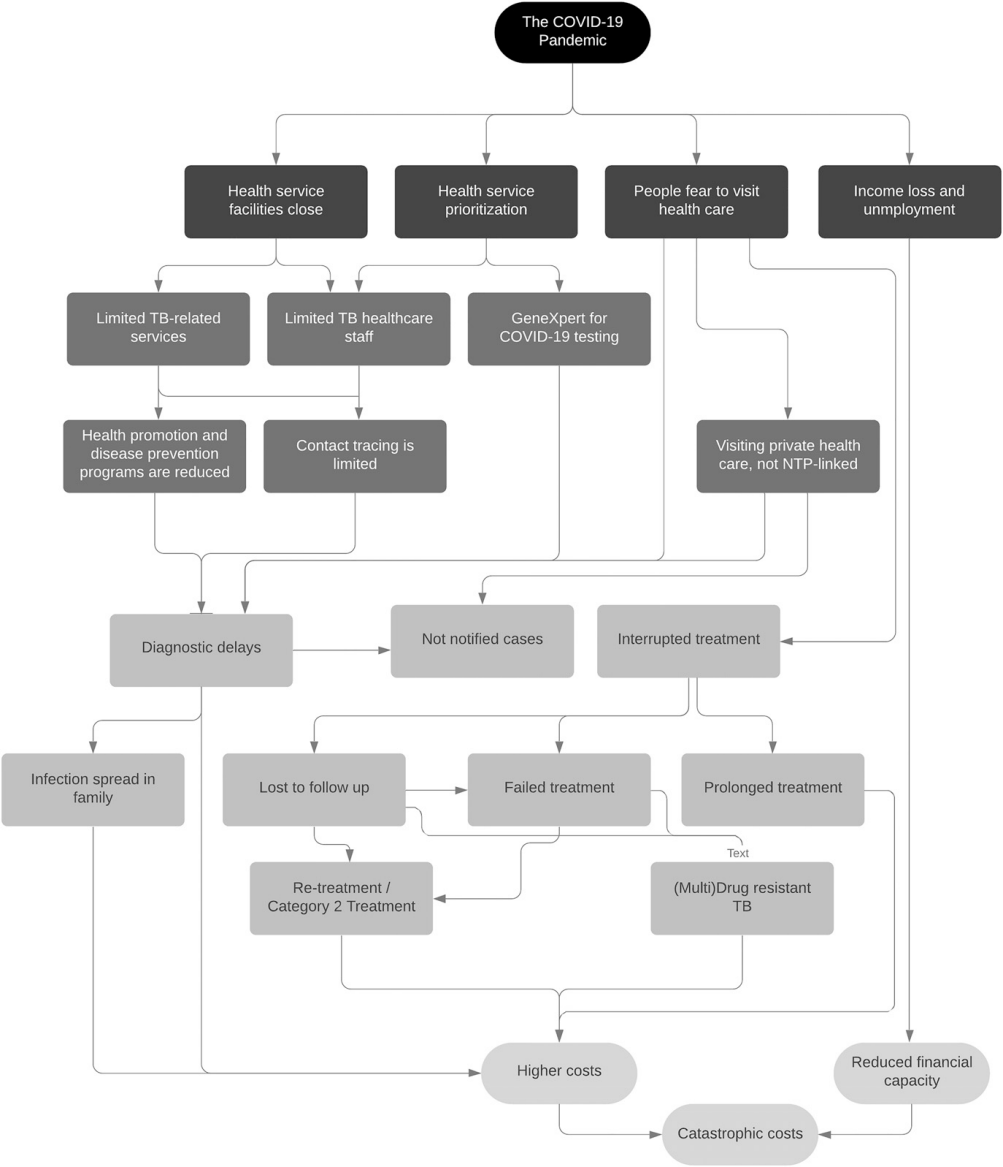
Impact of the COVID-19 pandemic on tuberculosis (TB)–related catastrophic costs.

The COVID-19 pandemic has affected TB control programs and the incidence of catastrophic costs by distorting the health system at different levels. Figure 1 illustrates potential pathways by which COVID-19 can impact TB care-seeking, treatment, and household income and cost incurred as a result of the disease. In general, the most noticeable effect is the prioritization for COVID-19–related healthcare services above other health problems. The prioritization has also resulted in a role switch of healthcare staff to COVID-19–related services that eventually have reduced the number of consultation services and the delivery of TB-related programs in either healthcare facility or community. The WHO reported that 122 countries have partially or entirely disrupted healthcare services for noncommunicable diseases.^29^ This could also reduce healthcare service availability for TB, as many TB-related resources are designated to COVID-19 services.^30,31^

In the primary care level, the pandemic could affect most health promotion and disease prevention programs. When physical distancing measures are promoted, primary healthcare facilities should reduce TB-related health promotion programs, which are delivered in groups. Contact tracing has also been affected because of a much more limited number of healthcare staff to do such program including TB preventive therapy that could increase the likelihood of secondary cases in households. Most TB-related services are also closed.^8^ In many LMICs, where healthcare resources are scarce, the severe reduction of TB-related healthcare services is likely to increase diagnostic delays and decrease the TB notifications.

In some countries, TB-related services are provided in secondary care–level facilities. MDR TB services, including the diagnostic procedure, are also often delivered in hospital. With an increasing volume of COVID-19 cases in hospitals, TB-related services and its healthcare staff are reduced—all eventually interrupting TB services in hospitals and likely to increase patients’ diagnostic delays.

One of modalities to reduce TB diagnostic delay is the rapid GeneXpert device. However, because of the pandemic and the vast need to identify SARS-CoV-2 virus, some countries encourage the use of GeneXpert to test suspected COVID-19 patients. For COVID-19 control, this initiative boosts the COVID-19 testing scale but also reduces the TB test capacity, particularly for countries that rely heavily on the GeneXpert device (Cepheid, Sunnyvale, CA). It would, therefore, hinder the identification of TB cases and increase its diagnostic delay.

The diagnostic delay and not notified cases are also aggravated by patient avoidance of healthcare services, in particular public healthcare services that are linked to the National Tuberculosis Program (NTP) and now commonly designated for COVID-19 services. Avoidance comes from a real concern that visiting such healthcare services may increase the risk of being infected by COVID-19.^32^ It may lead people to seek care with private healthcare providers that are not designated as COVID-19–related services and are not linked to the NTP. This preference could increase and, together with potential diagnostic delay, eventually may result in much higher pre-diagnostic costs incurred by suspected TB patients and their households.^33^

More than 50% of TB patients in Kenya and India are in fear of contracting COVID-19 at a health facility.^8^ Their avoidance of the already reduced TB-related services may lead to a higher number of interrupted TB treatments or even to patients being lost to follow-up. Patients who have interrupted TB treatment usually face prolonged TB treatment. Patients who are lost to follow-up may face more severe symptoms, fail to complete their treatment, undergo category 2 TB treatment (i.e., TB treatment received by patients who had failed treatment, lost to follow-up, or recurrence), or even encounter (multi)drug-resistant TB. It would increase the risk of hospitalization and more expensive treatment; two conditions that are risk factors for catastrophic costs due to TB.^34^

For drug-resistant TB patients, the pandemic causes a much more complicated situation. They still need to visit a hospital, where such care is often delivered, with a much higher caution from either themselves or healthcare staff to prevent the COVID-19 infection. If a patients’ concern of contracting COVID-19 leads to dismissal from accessing MDR-TB treatment, the risk increases of a more severe condition, longer treatment period, hospitalization, or extensively drug-resistant TB. All will result in a higher cost and risk of experiencing catastrophic costs or impoverishment.

Despite the risk of increasing TB-related costs, the pandemic may have positive effects as well concerning certain costs. Tuberculosis patients and their families are known to spend unnecessary costs on diagnostic tests, consultation, and hospitalization.^35,36^ The fear of visiting healthcare facilities because of COVID-19 may reduce such unnecessary costs. The pandemic may also raise awareness of respiratory disease symptoms and encourage people to seek care for respiratory complaints. In some cases, this may reduce diagnostic delay of TB and prevent unnecessary costs in the pre-diagnostic phase.

The COVID-19 pandemic also caused an economic recession that significantly impacts household financial capacity because of the rise in unemployment rates and the decrease of income.^37^ The job loss, income reduction, and poverty are risk factors for catastrophic costs due to TB.^13^

Poor households that face unemployment and substantial income loss may be forced to buy cheaper, less nutritious meals.^38^ This could lead to poor nutrition in the long run. Poor nutrition is a risk factor for the development of TB and, if a patient receives TB treatment, it may negatively affect TB treatment.^39^ The treatment may be unsuccessful and need to be continued to a longer period. When the TB treatment is prolonged, it can increase the risk of catastrophic costs due to TB.

## COVID-19 AND SOCIAL PROTECTION

The impact of COVID-19 on populations and TB-related health systems, particularly in LMICs, requires a robust and comprehensive response that incorporates a long-term strategy. Achieving the WHO End TB Strategy targets require measures beyond providing high-quality healthcare services. These ambitious targets, which are supposed to be achieved in the next 15 years, require political commitment to provide adequate financial protection for TB patients and their affected households. Several studies reported that providing national health insurance within the UHC framework is not enough to achieve the target of eliminating the incidence of catastrophic costs.^23,40^ Setting up comprehensive social protection programs is critical.

However, social protection systems in many TB HBCs are suboptimal, and are at risk of further weakening due to the pandemic. It is very difficult for countries to build institutional capacity during the pandemic, which would result in a limited and delayed response.^41,42^ A global economic recession will retract economic growth and result in scarce financial resources. Governments may shift national budget priorities to economic development and may limit their budgets for health-related programs. While many high-income countries may start to provide economic stimulus packages, most TB HBCs often depend on the external funds from donors.^7^ Many TB HBCs and LMICs need help from higher income countries to mount a sustained response. This would involve a substantial increase of investments in the provision of UHC, strengthening the national healthcare system with a particular focus on primary-level health care, and the provision of social support to vulnerable population groups.

Many countries provide TB-related services in a vertical system, detached from their national healthcare system and health financing system. This is a common approach applied, particularly when the major funding comes from international donors. However, it also may result in a fragmented, ineffective system to combat TB. Tuberculosis-related social protection is also often provided in a TB-specific approach that targets people diagnosed with TB and their affected household in the form of cash transfers or food parcels.^43,44^ Many social protection programs are detached from each other.^44,45^

Nevertheless, this pandemic, which reduces fiscal capacity of countries, may remind them to develop innovative social protection interventions for TB-affected households that are integrated into other social protection schemes instead of having many programs which are not linked to each other.^46^ This approach should improve equitable access to TB-related services, from health promotion to rehabilitation. It should also reduce the high costs incurred for accessing TB services, and therefore improve TB treatment outcomes. The social protection strategy also requires a clear understanding of patients’ needs and the government’s resources. In some setting, the NTP and the national health insurance are fragmented, and the integration of both could help control TB and avoid unnecessary costs. Any financial support should also engage patients and civil society organizations with the effort of preventing much more severe impact of COVID-19 on TB control programs.

It is clear that the COVID-19 pandemic will affect the WHO targets to reduce TB incidence and death rate and to eliminate the incidence of catastrophic costs. It is plausible that the risk of facing catastrophic costs increases in several ways. In response, it is important that health and social protection systems should be strengthened, particularly in TB HBCs, to reduce the devastating impact of the pandemic for TB-affected households. It is also important as part of strategies to attain the target of eliminating the TB burden worldwide. International support is required for countries that lack robust health and social protection systems.
